# Luteolin promotes healthy aging in adult females but shortens lifespan after embryonic exposure in *Drosophila melanogaster*

**DOI:** 10.3389/fnut.2026.1909085

**Published:** 2026-08-20

**Authors:** Quanyong Wu, Boya Ouyang, Hui Cao, Min Zhang, Mohamed A. Farag, Thomas Efferth, Jesus Simal-Gandara, Jianbo Xiao

**Affiliations:** 1 Nutrition and Bromatology Group, Department of Analytical Chemistry and Food Science, Instituto de Agroecoloxía e Alimentación (IAA), Universidade de Vigo, Vigo, Spain; 2 College of Oceanology and Food Science, Quanzhou Normal University, Quanzhou, China; 3 Pharmacognosy Department, Faculty of Pharmacy, Cairo University, Cairo, Egypt; 4 Department of Pharmaceutical Biology, Institute of Pharmaceutiocal and Biomedical Sciences, Johannes Gutenberg University, Mainz, Germany; 5 Research Group on Food, Nutritional Biochemistry and Health, Universidad Europea del Atlántico, Santander, Spain

**Keywords:** *Drosophila melanogaster*, epigenetic regulation, healthy aging, intervention timing, luteolin, metabolic remodeling

## Abstract

**Introduction:**

Luteolin is a dietary flavonoid widely distributed in fruits, vegetables, and medicinal plants and is recognized for its antioxidant and health-promoting properties. However, its effects on physiological aging and the influence of developmental stage and biological sex remain poorly understood.

**Methods:**

In this study, *Drosophila melanogaster* was exposed to luteolin during either adulthood or embryonic development and evaluated for lifespan, healthspan, metabolic regulation, and aging-related molecular changes.

**Results:**

Adult supplementation exerted pronounced female-specific benefits, increasing median lifespan by 11.6%, improving locomotor activity and stress resistance, with little effect on body weight, whereas males showed little response. Notably, short-term supplementation during early adulthood produced sustained protective effects later in life. Mechanistically, luteolin enhanced antioxidant defenses, suppressed IIS/PI3K–mTOR signaling, activated AMPK/MAPK pathways, promoted autophagy and immune homeostasis, and induced metabolic remodeling associated with reduced mitochondrial stress and favorable histone modifications. In contrast, embryonic exposure shortened lifespan and disrupted endocrine and metabolic homeostasis, as evidenced by elevated juvenile hormone levels, reduced catalase activity, and dysregulated IIS signaling.

**Discussion:**

These findings suggest that the biological effects of luteolin on aging are highly dependent on developmental stage and biological sex in *Drosophila*. The beneficial effects observed in adult females were associated with coordinated changes in nutrient-sensing pathways, metabolism, mitochondrial function, and epigenetic regulation, whereas developmental exposure produced adverse outcomes. Collectively, this study provides new insights into the context-dependent actions of dietary flavonoids in *Drosophila* and highlights the importance of developmental stage and biological sex when evaluating nutritional interventions for healthy aging.

## 1 Introduction

Aging is a multifactorial biological process characterized by progressive declines in physiological function and resilience, driven by dysregulation of nutrient sensing, loss of proteostasis, mitochondrial dysfunction, and epigenetic alterations. Advances in geroscience have challenged the traditional view of aging as an inevitable and irreversible process, demonstrating instead that aging trajectories can be modified through targeted interventions that delay functional decline and extend healthspan (1). Among the most extensively studied approaches are dietary and pharmacological interventions targeting conserved longevity pathways, including caloric restriction mimetics, activators of sirtuins and AMP-activated protein kinase (AMPK), and inhibitors of mechanistic target of rapamycin complex 1 (mTORC1) signaling (2). In this context, naturally occurring dietary polyphenols have attracted considerable attention because of their favorable safety profiles and pleiotropic biological activities. Their proposed anti-aging mechanisms include attenuation of oxidative stress and inflammation, preservation of mitochondrial homeostasis, and promotion of autophagy (3).

Luteolin is a naturally occurring flavone widely distributed in herbs, vegetables, and spices, including oregano, celery, and fennel. It has been extensively studied for its antioxidant, anti-inflammatory, and cytoprotective properties (4). Current research has primarily focused on its anticancer activity and protective effects on multiple organs, including the skin, nervous system, and liver (5). Despite these promising biological activities, the therapeutic application of luteolin remains challenging because of its poor aqueous solubility, low oral bioavailability, and rapid phase II metabolism, which substantially limits its systemic exposure and pharmacological efficacy *in vivo* (6). Consequently, a clearer understanding of the biological contexts in which luteolin exerts robust beneficial effects is essential for its rational development as a dietary or pharmacological intervention. Notably, luteolin has been reported to modulate several evolutionarily conserved signaling pathways closely linked to aging, including insulin/insulin-like growth factor signaling (IIS), target of rapamycin (TOR), and oxidative stress response pathways (7). Despite these observations, current evidence is largely derived from disease models or stress-induced injury models, whereas its effects on physiological aging under normal conditions remain poorly defined. Furthermore, whether the biological effects of luteolin depend on the timing of exposure during the life course or differ between males and females has not been systematically investigated. Therefore, whether luteolin can truly modulate the natural aging process and promote healthy aging remains largely unexplored.

*Drosophila melanogaster* is a well-established model organism for aging research owing to its short lifespan, genetic tractability, and conservation of key nutrient-sensing and longevity-associated pathways. Moreover, aging-related phenotypes such as lifespan, locomotor performance, and stress resistance can be readily quantified in this model (8). An important feature of aging biology is the existence of marked sex-specific differences in lifespan regulation, metabolism, and aging-related physiological processes across both humans and model organisms (9). In *Drosophila*, sexual dimorphism has been widely documented in lifespan trajectories, energy metabolism, and the trade-off between reproduction and longevity (10). In addition, developmental stages represent distinct windows of sensitivity to nutritional and environmental cues, with early-life interventions often producing long-lasting effects on adult physiology and lifespan (11). These characteristics make *Drosophila* an ideal system for investigating the stage- and sex-dependent effects of dietary interventions on aging.

Importantly, increasing evidence suggests that the biological effects of dietary polyphenols are highly context dependent (12). Their efficacy is influenced by multiple biological factors, including developmental stage, sex, physiological status, dosage, and metabolic context. For example, early-life nutritional interventions can induce persistent physiological programming that differs fundamentally from responses to interventions initiated during adulthood, while sex-specific differences in endocrine regulation, nutrient sensing, and energy metabolism further contribute to divergent aging phenotypes (13, 14). Despite growing recognition of these context-dependent responses, most previous studies have investigated luteolin under a single intervention stage, in only one sex, or within disease-related models (15, 16). Consequently, it’s difficult to determine whether its reported anti-aging effects are generalizable to physiological aging. Addressing these unresolved questions is essential for defining the biological conditions under which luteolin promotes healthy aging and for guiding its rational application as a dietary antiaging intervention.

In this study, we aimed to determine whether the anti-aging effects of luteolin are dependent on biological context, with particular emphasis on sex and developmental stage. *Drosophila melanogaster* was exposed to luteolin during either embryonic development or adulthood, and lifespan, body weight, locomotor activity, stress resistance, and other healthspan-related traits were systematically evaluated. Furthermore, we examined the potential molecular mechanisms underlying the observed effects, focusing on nutrient-sensing pathways, oxidative stress responses, autophagy, metabolism, mitochondrial function, and epigenetic regulation. By integrating stage-specific, sex-specific, and mechanistic analyses within a single experimental framework, this study provides a more comprehensive understanding of the context-dependent actions of luteolin on aging and offers new insights into the rational use of dietary flavonoids for healthy aging.

## 2 Materials and methods

### 2.1 Chemicals and reagents

Luteolin (purity ≥ 98.0%) was obtained from Aladdin Industrial Corporation (Shanghai, China). Corn flour (food-grade, *Gofio la piña*), sucrose (food-grade, *Auchan*), and yeast (food-grade, *Saf*-instant) were purchased from a local supermarket in Vigo, Spain. Agar powder was purchased from Beijing Solable Technology Co., Ltd. (Beijing, China). The superoxide dismutase and catalase kits were provided by Nanjing Jiancheng Bioengineering Institute (Jiangsu, China). Juvenile hormone and ecdysone Enzyme-linked Immunosorbent assay (ELISA) kits were provided by Jiangsu Jingmei Biological Technology Co., Ltd. (Jiangsu, China). Trizol reagent was purchased from Thermo Fisher Scientific Inc. (Massachusetts, USA). The antibodies were purchased and used at the indicated dilutions for Western blotting, primary antibody included rabbit anti-Histone H3, rabbit anti-H3K4me3, rabbit anti-H3K9me3, rabbit anti-H3K27me3, rabbit anti-H3K36me3, rabbit anti-H3K9ac, and rabbit anti-H3K27ac, all were obtained from Abcam (Cambridge, UK), and second antibody, goat anti-rabbit IgG, were obtained from Proteintech Group, Inc. (Cell Signaling, USA).

### 2.2 Fly strains and husbandry

The Oregon wild-type *Drosophila* strain was kindly provided by Prof. Armando Caballero Rúa from the University of Vigo (Vigo, Spain). The flies were maintained on a modified standard medium as previously described, consisting of 6.5% (w/v) cornmeal, 4% (w/v) sucrose, 1% (w/v) agar, and 1% (w/v) yeast (17). All flies were reared in temperature- and humidity-controlled incubators at 25 °C with 60% relative humidity under a 12 h light/12 h dark cycle.

### 2.3 Experimental flies and medium

New emergence flies (<8 h) were collected from standard cornmeal medium to ensure population synchrony. Under light CO₂ anesthesia, flies were separated by gender and allocated to different diets: Normal control medium (NC; standard medium), solvent control medium (SC; 1% v/v ethanol added to standard medium at 60 °C). Based on previous research reports, 50 μg/mL luteolin was chosen as the concentration for this experiment (LUT50; 50 μg/mL luteolin dissolved in ethanol added to standard medium at 60 °C) (18, 19). The males, eclosed from normal culture medium within 8 h, exposed to SC for 10 days were crossed on NC medium with females treated similarly for 10 days with either SC or LUT50 (3 males and 3 females per cross) to generate F1 progeny. Newly eclosed F1 adults were maintained on NC medium.

### 2.4 Embryonic development

Adult flies that eclosed on standard medium were transferred to NC, SC or LUT50 medium for egg laying (3 males and 3 females per vial, 3 vials per group). On day 5, the parental flies were removed. From the onset of pupation and emergence, the numbers of pupae and eclosed adults were recorded daily for 5 consecutive days. Newly eclosed flies were counted and immediately transferred to standard medium for lifespan assays.

### 2.5 Lifespan and health assays

For lifespan analysis, 60 flies (20 per vial, three vials per group) were maintained under each treatment. The mortality was recorded daily, and flies were transferred to a fresh diet every 3 days. The health status was assessed by measuring body weight, locomotor activity, and oxidative stress resistance. Body weight changes were monitored for 30 days by weighing the average weight of flies per vial every 5 days. The locomotor activity was evaluated after 10 days of intervention using a negative geotaxis assay (20). Briefly, groups of 10 flies were transferred into 50 mL centrifuge tubes sealed with cotton plugs, tapped to the bottom, and allowed to climb for 30 s. The tube was divided into three zones: top (>4 cm), middle (2–4 cm), and bottom (<2 cm). The climbing score was calculated as 10 × (n_top) + 5 × (n_middle) + 1 × (n_bottom). Each vial was tested three times, with three biological replicates per group. Oxidative stress resistance was assessed by hydrogen peroxide exposure. Groups of 10 flies were starved for 2 h, then transferred into 50 mL centrifuge tubes containing filter paper soaked with 30% H₂O₂ and 6% sucrose solution. The mortality was recorded every 2 h until all flies died, with three biological replicates per group.

### 2.6 Antioxidant enzyme assay

Superoxide dismutase (Sod) and catalase (Cat) activities in *Drosophila* were determined using commercial assay kits according to the instructions of the manufacturer. Briefly, 10 flies, which were as one sample, were homogenized in 900 μL pre-cooled PBS buffer (pH 7.4) and centrifuged at 12,000 × g for 10 min at 4 °C. The supernatant was collected and stored at −80 °C for subsequent enzyme activity assay, three biological replicates per group. The total protein concentration was quantified using the Bradford assay (Thermo Fisher Scientific) and used to normalize enzyme activities.

### 2.7 Hormone content determination

The juvenile hormone (JH) and ecdysone levels were measured using commercial ELISA kits according to the protocols of the manufacturers. Briefly, homogenates were prepared as described in section 2.6. and added to antibody-precoated microplates (JH and ecdysone) and incubated at 37 °C for 30 min, followed by five washes. The enzyme-labeled reagent was then added and incubated under the same conditions for 30 min. After five additional washes, colorimetric substrate was applied and the plates were incubated at 37 °C in the dark for 10 min, after which the reaction was terminated with stop solution. Absorbance was measured at 450 nm using an Epoch BioTek microplate reader, and hormone concentrations were determined from standard curves generated with serial dilutions of the supplied standards. The total protein content was quantified separately using a Bradford assay kit (Thermo Fisher Scientific).

### 2.8 LC–MS assay

Ten flies were homogenized in 800 μL acetonitrile: methanol: water (2:2:1, v/v/v) containing 300 μM L-methionine sulfone. After centrifugation (14,000 × g, 10 min, 4 °C), supernatants were dried under vacuum and reconstituted in 100 μL acetonitrile: methanol: water (2:2:1, v/v/v). UHPLC was performed on a Thermo UltiMate 3,000 system with an Agilent InfinityLab Poroshell 120 HILIC-Z column (2.1 × 100 mm, 2.7 μm) at 25 °C. The mobile phases were water with 10 mM ammonium acetate, pH 9.0 or water with 10 mM ammonium (positive ion mode) and B mobile phases were acetonitrile with 10 mM ammonium acetate, pH 9.0 or acetonitrile with 10 mM ammonium acetate (positive ion mode), with a flow rate of 0.3 mL/min and a gradient as follows: 0 min: 95% B, 10 min: 50% B, 13 min: 95% B, 13–16 min: re-equilibration at 95% B or 0 min: 95% B, 5 min: 50% B, 8 min: 20% B, 10 min: 5% B, 13 min: 95% B, 13–16 min: re-equilibration at 95% B (positive ion mode). The injection volume was 5 μL.

Detection was carried out on a Thermo TSQ Quantis triple quadrupole mass spectrometer with H-ESI in positive (3,500 V) or negative (2,500 V) ion modes. The source parameters were: sheath gas 50 Arb, auxiliary gas 10 Arb, ion transfer tube 325 °C, vaporizer 350 °C. The data was acquired in SRM mode with transitions optimized using authentic standards, including: AMP (*m/z* 346 to 134), ADP (*m/z* 426 to 328), R5P (*m/z* 229 to 97), GSH (*m/z* 306 to 143), DF6P (*m/z* 259 to 97), S7P (*m/z* 289 to 199), ATP (*m/z* 506 to 408), NADPH (*m/z* 744 to 426), G6P (*m/z* 259 to 199), E4P (*m/z* 199 to 97) DHAP (*m/z* 169 to 97), NADP^+^ (*m/z* 742 to 408), GSSG (*m/z* 611 to 306), F16BP (*m/z* 339 to 97) in negative ion mode, and SAM (*m/z* 400 to 250) and SAH (*m/z* 386 to 136.5) in positive ion mode. The quantification was performed using Xcalibur software (Thermo Fisher Scientific, Waltham, MA, USA) with 300 μM L-methionine sulfone as an internal standard. All SRM transitions were established and optimized using authentic standards prior to sample analysis. Peak integration and quantification were performed using Xcalibur software, and the peak area of each metabolite was normalized to the internal standard (L-methionine sulfone) before statistical analysis. To minimize analytical variability, all samples were prepared using an identical extraction protocol and analyzed under the same instrumental conditions within a single analytical batch.

### 2.9 GC–MS assay

Ten flies were homogenized in 800 μL of acetonitrile: methanol: water (2:2:1, v/v/v) containing 20 μM norvaline as internal standard. The homogenates were incubated at −20 °C to precipitate proteins, centrifuged (14,000 × g, 15 min, 4 °C), and 500 μL of the supernatant was dried under vacuum. The residues were reconstituted in 50 μL DMF at 37 °C for 90 min, derivatized with 50 μL MTBSTFA at 60 °C for 30 min and centrifuged before GC–MS analysis.

The GC–MS analysis was performed on a Thermo Trace 1,300 coupled with an ISQ 7000 using an Rxi-5 ms column (30 m × 0.25 mm, 0.25 μm; Restek). Helium was used as carrier gas (1.5 mL/min). The samples were injected in split mode (1:200–1:500) at 300 °C. The oven program was: 110 °C (4 min), ramped to 215 °C at 15 °C/min, to 260 °C at 5 °C/min, and to 310 °C at 25 °C/min (3 min hold). The temperature of inlet and GC–MS interface were maintained at 280 °C and 230 °C, respectively. The data was acquired in SIM mode for targeted metabolite quantification, including citrate (*m/z* 431 and 459), *α*-ketoglutarate (*m/z* 346), succinate (*m/z* 289), fumarate (*m/z* 287), malate (*m/z* 391 and 419), serine (*m/z* 362 and 390), glycine (*m/z* 218 and 246), methionine (*m/z* 218 and 320), cysteine (*m/z* 406), phenylalanine (*m/z* 336), GAP (*m/z* 383), 3PG (*m/z* 585), PEP (*m/z* 453), pyruvate (*m/z* 259), lactate (*m/z* 233 and 261), valine (*m/z* 260), alanine (*m/z* 232 and 260), glutamine (*m/z* 357 and 431), glutamate (*m/z* 330 and 432), aspartate (*m/z* 302, 390 and 418). All target metabolites were identified and quantified based on authentic standards under optimized SIM conditions. Peak integration and quantification were performed using Xcalibur software (Thermo Fisher Scientific), and the peak area of each metabolite was normalized to the internal standard (20 μM norvaline) prior to statistical analysis. To minimize analytical variability, all samples were processed using identical extraction, derivatization, and GC–MS analytical procedures and analyzed under the same instrumental conditions within a single analytical batch.

### 2.10 RNA-seq

The total RNA was extracted from whole flies using Trizol reagent. The RNA quality was assessed with the Agilent Bioanalyzer 2,100 (Agilent Technologies, CA, USA), and samples with RIN > 7.0 were used for library preparation. The RNA-seq libraries were generated by Beijing Novogene Technology Co., Ltd. (Beijing, China). Briefly, mRNA was enriched with poly-T oligo magnetic beads, fragmented, and reverse transcribed to cDNA, followed by end repair, adaptor ligation, size selection (370–420 bp), and PCR amplification. The library quality was evaluated on the Agilent Bioanalyzer 2,100. Sequencing was performed on an Illumina NovaSeq 6,000 platform with 150 bp paired end reads. Clustering was conducted on a cBot system using the TruSeq PE Cluster Kit v3-cBot-HS (Illumina) according to the manufacturer’s instructions. Raw sequencing reads were subjected to quality control by Beijing Novogene Technology Co., Ltd. using standard quality assessment procedures, including adaptor trimming and removal of low-quality reads. Only high-quality clean reads were retained for downstream transcriptomic analysis. Read mapping, transcript quantification, and differential expression analysis were performed using the standard Novogene RNA-seq analysis pipeline.

### 2.11 Quantitative real-time PCR

Total RNA was extracted from whole *D. melanogaster* using TRIzol reagent (Thermo Fisher Scientific, USA). The RNA concentration and quality were assessed with a NanoDrop spectrophotometer (Thermo Fisher Scientific, USA), and first-strand cDNA was synthesized using a reverse transcription kit (Thermo Fisher Scientific, USA).

qRT-PCR was performed with SYBR Green Master Mix (Beyotime Biotechnology, China) on a CFX96 Connect™ Real-Time PCR System (Bio-Rad, USA). *Rp49* served as the internal reference gene. Relative expression levels were calculated using the 2^–ΔΔCt^ method. Each reaction was run in triplicate with three biological replicates. Primer sequences (synthesized by Sangon Biotech, Shanghai, China) are listed in Table 1.

**Table 1:** List of primers for qRT-PCR assays.

Genes	Forward primer	Reverse primer
*Sod1*	TCTTCGAACAGGAGAGCAGC	GCCATACGGATTGAAGTGCG
*Sod2*	AAGCTGCCCTACGACTATGC	GAGGGACGCACGTTCTTGTA
*Cat*	TTCCTGGATGAGATGTCGCACT	TTCTGGGTFTFAATFAAFCTGG
*Jafrac1*	CGATGAGGAGACTGGCATCC	TTGTCGGTGTACTGGAAGGC
*Irp-1A*	GCAATTTCGAGGGTCGCATC	CTGAATCTCACTGCGCGTTG
*Irp-1B*	TCATCGTTCCAACCTGGTGG	CAGGGCGATGTTGTACACCT
*CncC*	GGGATCCCCATAAGCAGACG	AGTCCTTGGATAGAGCGGGT
*chico*	AAATTCAGCAAAGCCAGCCG	TATGCGAGACGACGACCTTG
*InR*	CAGTGGTTCGGCTGGTAGTT	CGTTAGGATGGTGGCCTGTT
*Ilp2*	AATTCCCTGGCTGAAGTCCG	CTGACCACGGAGCAGTACTC
*AMPKα*	GGCCATCGCTTACCATCTGA	ATCGATGATTGGGGAACGGG
*Lkb1*	ATACGCCACGACACTTGGTT	ACCGTGGAGTTGCGGTATTT
*CaMKII*	CTCGCAGTGCTCTTCAGCTA	TTTGCAGTTTTTACCAAGAAC
*Pi3K92E*	CCTAATCTGCCTGTTGCCCA	ACTGAGTCGCTTCGTTTCGT
*Thor*	ACCCTCTACTCCACCACTCC	GGAGTTTGGCTCAATGGGGA
*Nprl2*	GAAATACGTGGCCTTGCGTC	AATCTCTGGCAGATCGCTCG
*Tsc1*	CCAGCATACAATCAAGCCGC	GGCTCCATCTCCTTCCATCG
*rl*	ATGGTTGTGTCTGCGGATGA	GTTTGGTGTTCAAAGGGCGA
*bsk*	CACCAACACTACACCGTCGA	TCGCTTGGCATGGGTTACAT
*p38a*	ATCCCGCTAATGGATCGCTG	CCGGCGCTGTGGATATACTT
*S6kII*	AGGAGAGAAGGGGCTACTGG	AAGCTACCTTCGCCCAGAAC
*Dsor1*	ATACGCAAGGCGGAATTGGA	TTGGGCGACGTATTACGCTT
*Hsp70*	AGCCGTGCCAGGTTTG	CGTTCGCCCTCATACA
*Hsf*	CATTAGCTCGCACAACGTCG	ACTTTGGGCCATGGGAGAAG
*Atf-2*	CGGAGACATGGATCACCTGG	GATAGCAGGATACGTCGCCC
*Atg1*	GCCAGCTCCATCGAAAATAACC	GCGGCGCAGCAGGCACAG
*Atg5*	GCCCCTGCGACTTCACTATCC	CCATTAAATCGGCCAAACTCTTCT
*Atg8a*	CCAATACAAGGAGGAGCAC	AGGAAGTAGAACTGACCGAC
*Atg8b*	AATGTGATCCCACCGACATC	TTGAGCGAGTAGTGCCAATG
*p53*	GGAATTGACCACGGAACCCA	ATCCAAAGAGACTTGGGAGG
*Dif*	AACCAAACACAATCGCCGTG	ATGGGTAGGACTACAGCCGT
*Dredd*	CGGCCATTTGAAGTCCAACG	ATGGACAAGCTTCTCCTGGC
*Relish*	GGCCATCCAGACGATACAGG	CCATACCCAGCAAAGGTCGT
*Def*	CGCTTTTGCTCTGCTTGCTTGC	TAGGTCGCATGTGGCTCGCTTC
*Drs*	TCCTGATGCTGGTGGTCCTG	CTCCTCCTTGCACACACGAC
*Sir2*	AACCAGGTGCCAGACACTAC	GTTGAGGCCAGAATTTCCGC
*nej*	TGCGGCAGGAAGTACACATT	TCTATTTGAGGCGACACCGT
*HDAC4*	CGAATGGCACAACGACAACG	GTTCGGTTCGGATGCTGTCT
*Ash1*	ATCTCCAAGAGCTATGCGCC	CCCAACCATCTTGGCCTGAT
*Set1*	ATGGCAAAACTTCAACCGCC	GGCGGTGCATTTCCAAAAGT
*Kr-h1*	ACGAGGAGCCCACAGATTTG	ACCTGGTCATCGTTGTCGTC
*Gce*	GTACATGGGCTATGGACCGG	GTGGTGAATGATGCGGATGC
*Foxo*	GCGCCTCTACTTTGATAGGAC	TATTGATGTCCAGCAGGCCG
*Rp49*	ACAGGCCCAAGATCGTGAAG	GCGCTTGTTCGATCCGTAAC

All primers were designed based on *D. melanogaster* mRNA sequences obtained from NCBI or FlyBase and synthesized by Sangon Biotech (Shanghai, China).

### 2.12 Western blotting

The total protein was extracted from whole *D. melanogaster* using RIPA lysis buffer (Beyotime, China) with protease inhibitors. The homogenates were centrifuged (14,000 × g, 15 min, 4 °C), and protein concentrations were determined using Bradford assay. Equal amounts of protein (30 μg) were resolved by 12% SDS-PAGE and transferred onto PVDF membranes (Beyotime, China). The membranes were blocked with 5% BSA in TBST, incubated overnight at 4 °C with primary antibodies (1:4000), and then with secondary antibodies (1:5000) for 1.5 h at room temperature. All antibodies were diluted with 5% BSA in TBST. Signals were detected using ECL reagent (Beyotime, China) and a C-DiGit® Blot Scanner (LI-COR, USA). Band intensities were quantified using ImageJ, and protein expression was normalized to H3.

### 2.13 Statistical analysis

Statistical analyses were performed using GraphPad Prism 8.0.1 (GraphPad Software, San Diego, CA, USA) and R software (version 4.2.1; R Core Team, Vienna, Austria). All experiments were independently performed in at least three biological replicates, and data are presented as mean ± standard error (SEM) unless otherwise indicated. The group differences were evaluated by student’s t-test, with *p* < 0.05 considered statistically significant. The lifespan was analyzed using Kaplan–Meier survival curves and compared by the log-rank (Mantel–Cox) test. The differential gene expression analysis was conducted with the DESeq2 package (v1.20.0) in R, which applies to a negative binomial distribution model. *p*-values were adjusted using the Benjamini–Hochberg method, and genes with an adjusted *p* < 0.05 were considered differentially expressed.

## 3 Results

### 3.1 Luteolin extends lifespan and improves healthspan-related traits in adult female *Drosophila melanogaster*

**Figure 1:**
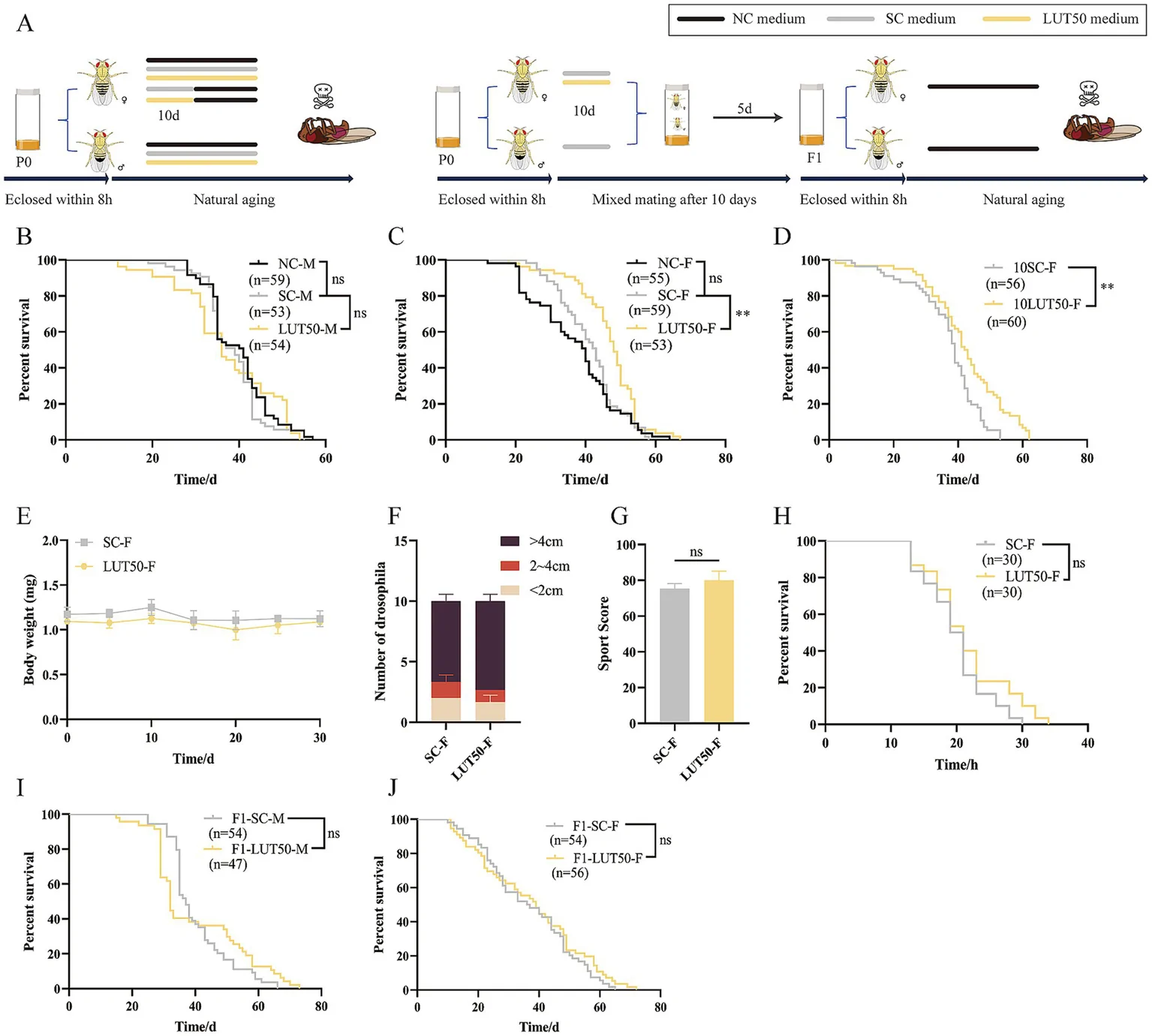
Effects of luteolin intervention during adulthood on lifespan and health in *D. melanogaster* under natural aging. **(A)** Newly eclosed males and females were separated, reared on specified diets, and monitored daily for mortality. In parallel, flies were subjected to 10-day dietary intervention, mated to produce offspring, and progeny were reared on standard diet with lifespan recorded. Each group contained 60 flies (20/vial, 3 vials), with food changed every 3 days. **(B)** Lifespan of male flies under natural aging; **(C)** lifespan of female flies under natural aging; **(D)** lifespan of female flies after 10-day luteolin treatment; **(E)** body weight changes of female flies within 30 days; **(F)** climbing height and **(G)** locomotor score of 10-day-old female flies; **(H)** survival of 10-day-old female flies under oxidative stress. Lifespan of progeny derived from luteolin-treated 10-day-old females: **(I)** male and **(J)** female offspring. Kaplan–Meier survival curves were analyzed using the log-rank (Mantel–Cox) test. Statistical significance is indicated as follows: ns, not significant; *p < 0.05; **p < 0.01; ***p < 0.001; ****p < 0.0001.

Initial experiments were conducted to evaluate the effects of luteolin supplementation and intervention duration on lifespan in male and female *D. melanogaster*, as well as potential effects on offspring longevity. As illustrated in Figure 1A, male and female flies within 8 h of eclosion were separated and subjected to either continuous or 10-day luteolin intervention. Survival was monitored daily throughout adulthood, and the lifespan of offspring derived from females receiving the 10-day intervention was subsequently assessed.

Compared with the SC group, no significant differences in lifespan were observed in either male or female flies of the NC group. Luteolin supplementation (LUT50) did not significantly affect lifespan in males. In contrast, female flies exhibited a significant lifespan extension, with median lifespan increasing by 11.6% (*p* < 0.01) and maximum lifespan also being significantly increased (Figures 1B,C). Notably, a 10-day LUT50 intervention during early adulthood was sufficient to produce long-lasting benefits, significantly extending lifespan in female flies (*p* < 0.01) and increasing maximum lifespan by 17.0% relative to the SC group (Figure 1D). To further evaluate healthspan-related outcomes, body weight, locomotor performance, and oxidative stress resistance were assessed following the 10-day intervention. Compared with the SC group, LUT50-treated females showed no significant differences in body weight throughout the experimental period, although a trend toward better body weight maintenance was observed at later time points. In addition, a greater proportion of flies climbed above 4 cm, locomotor performance was significantly improved, and survival under oxidative stress tended to be prolonged, although the latter did not reach statistical significance (Figures 1E–H).

We further investigated whether the lifespan-promoting effects of luteolin could be transmitted to subsequent generations. However, no significant differences in lifespan were observed in either female or male offspring derived from LUT50-treated females compared with the SC group (Figures 1I,J).

### 3.2 Transcriptomic analysis identifies pathways associated with luteolin treatment in adult female *Drosophila melanogaster*

**Figure 2:**
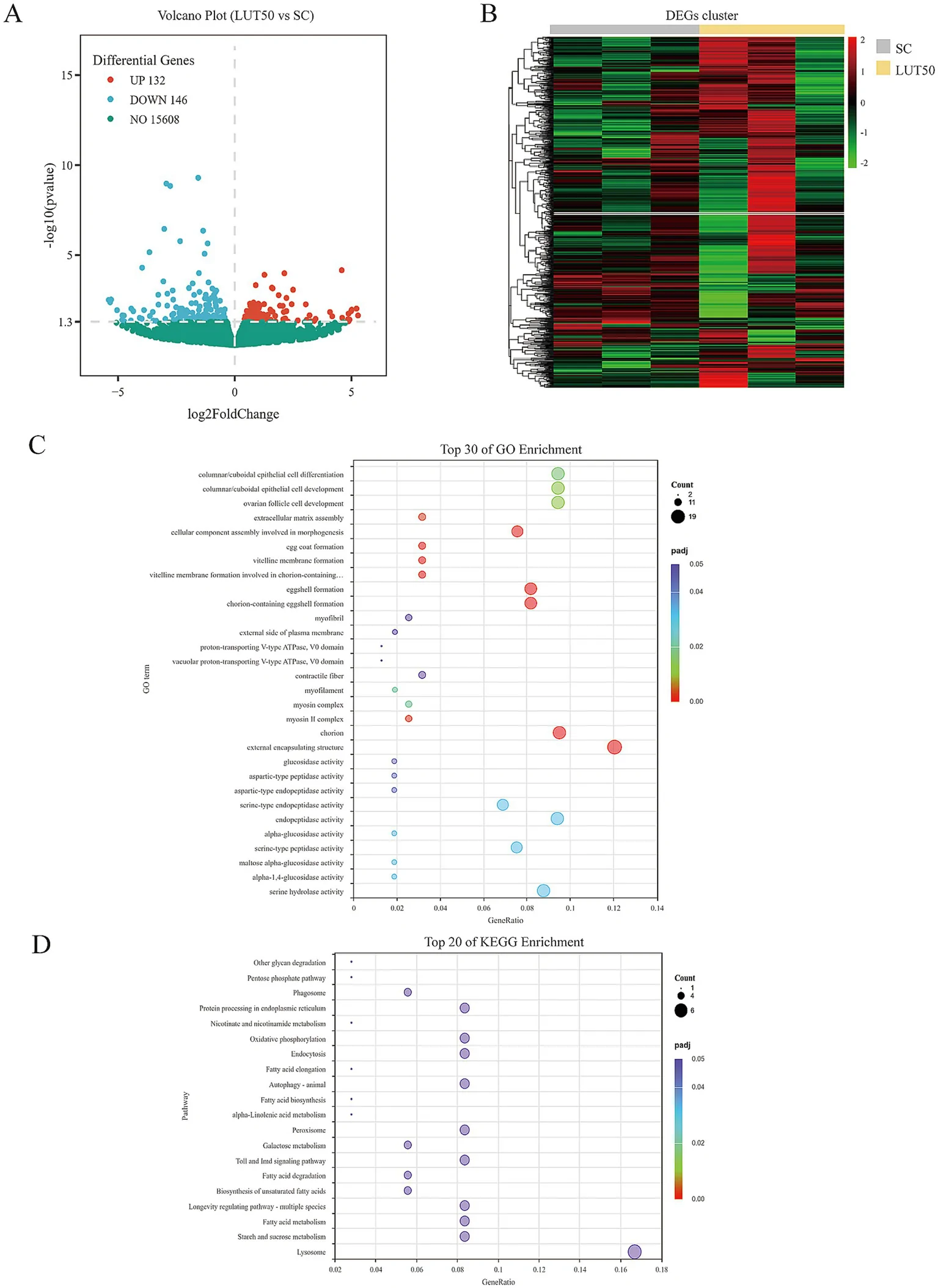
Transcriptional profiling of adult female *D. melanogaster* after 10-day luteolin intervention. **(A)** Volcano plot showing differentially expressed genes (DEGs) between SC and LUT50-treated females. **(B)** Hierarchical clustering heatmap illustrating distinct transcriptional patterns induced by luteolin treatment. **(C)** Gene ontology (GO) enrichment analysis of DEGs. **(D)** KEGG pathway enrichment analysis of DEGs.

Aging is a complex process involving multiple cellular molecules and signaling pathways (21). To investigate the molecular basis underlying the longevity-promoting effects of luteolin, transcriptomic analysis was performed in female flies following a 10-day LUT50 intervention. Compared with the SC group, LUT50 treatment resulted in 132 significantly upregulated and 146 significantly downregulated genes (Figure 2A). Hierarchical clustering analysis revealed a distinct transcriptional profile in LUT50-treated flies relative to the SC group (Figure 2B).

GO enrichment analysis showed that differentially expressed genes were primarily associated with oocyte-related processes, including eggshell formation, egg coat formation, chorion organization, external encapsulating structure, and vitelline membrane formation, as well as structural components such as contractile fibers and myosin complexes (Figure 2C). In addition, several enriched terms were related to metabolic enzyme activities, including glucosidase and serine-type endopeptidase activities.

KEGG pathway analysis further demonstrated significant enrichment of pathways involved in energy metabolism, including the pentose phosphate pathway, fatty acid metabolism, and oxidative phosphorylation. Pathways associated with cellular turnover and quality control, such as lysosome, autophagy–animal, phagosome, endocytosis, and protein processing in the endoplasmic reticulum, were also significantly affected. Moreover, immune- and longevity-related pathways, including Toll and Imd signaling and the longevity regulating pathway, were enriched following LUT50 treatment (Figure 2D). Together, these findings indicate that luteolin induces broad transcriptional changes involving metabolism, cellular turnover, autophagy, immune regulation, and longevity-associated pathways in adult female flies.

### 3.3 Key longevity-associated genes regulated by luteolin in adult female *Drosophila melanogaster*

To validate the transcriptomic findings and further characterize pathways associated with luteolin-mediated lifespan extension, the expression of key genes involved in oxidative stress responses, nutrient sensing, autophagy, and immunity was analyzed by qRT-PCR.

**Figure 3:**
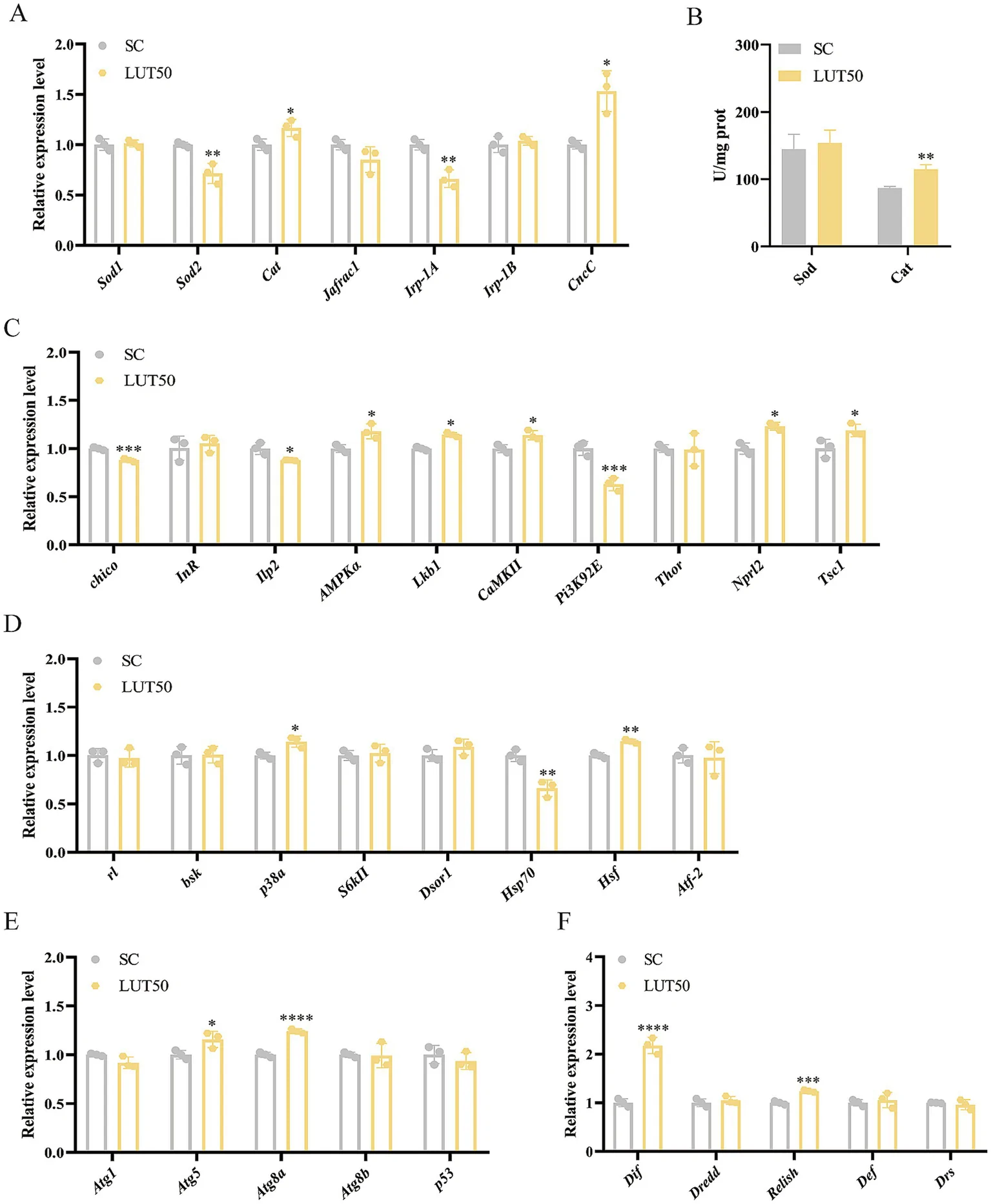
Expression of key genes in lifespan-associated signaling pathways of adult female *Drosophila* after 10-day luteolin intervention. **(A)** Antioxidant-related gene expression. **(B)** Enzymatic activities of Sod and Cat. **(C)** Expression of genes involved in nutrient-sensing pathways. **(D)** Expression of genes in the MAPK signaling pathway. **(E)** Expression of autophagy-related genes. **(F)** Expression of genes related to innate immune signaling.

Compared with the SC group, LUT50 treatment significantly downregulated *Sod2* and *Irp-1A* (*p* < 0.01), while significantly upregulating *Cat* and *CncC* (*p* < 0.05) (Figure 3A). Consistent with these transcriptional changes, catalase activity was significantly increased following LUT50 treatment (*p* < 0.01), whereas total Sod activity remained unchanged (Figure 3B). Genes associated with nutrient-sensing pathways were also markedly altered. LUT50 significantly reduced the expression of *chico* (*p* < 0.001), *Ilp2* (*p* < 0.05), *Pi3K92E* (*p* < 0.001), and *Hsp70* (*p* < 0.01), while increasing the expression of *AMPKα*, *Lkb1*, *CaMKII*, *Nprl2*, *Tsc1*, and *p38a* (*p* < 0.05), as well as *Hsf* (*p* < 0.01) (Figures 3C,D). In addition, LUT50 significantly upregulated the autophagy-related genes *Atg5* (*p* < 0.05) and *Atg8a* (*p* < 0.0001), together with the immune-related genes *Dif* (*p* < 0.0001) and *Relish* (*p* < 0.001) (Figures 3E,F). Collectively, these results demonstrate that LUT50 treatment is associated with coordinated transcriptional changes in antioxidant defense, nutrient-sensing, autophagy, and immune-related pathways in adult female flies.

### 3.4 Luteolin alters metabolic profiles and histone modifications in adult female *Drosophila melanogaster*

To further investigate metabolic and epigenetic changes associated with luteolin treatment, metabolic intermediates and histone modifications were analyzed in LUT50-treated female flies.

**Figure 4:**
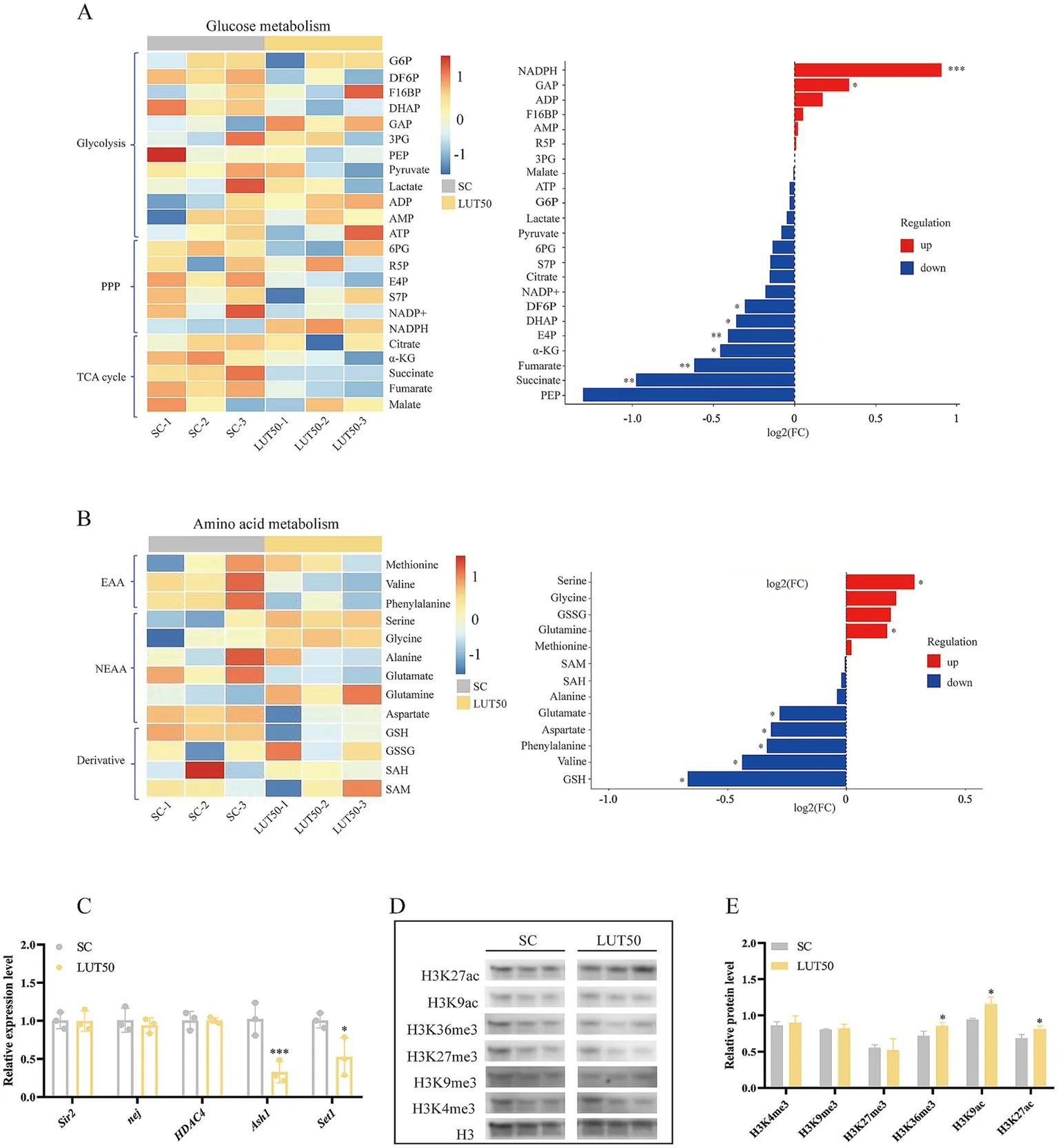
Changes in basal metabolism and histone modifications in adult female *D. melanogaster* after 10-day luteolin intervention. **(A)** Relative metabolite levels associated with glucose metabolism. **(B)** Relative metabolite levels associated with amino acid metabolism. **(C)** Expression of genes encoding histone methyltransferases and acetyltransferases. **(D)** Western blot analysis of selected histone modification types. **(E)** Relative abundance of histone modifications normalized to histone H3.

Significant alterations were observed in both glucose and amino acid metabolism following LUT50 intervention. Compared with the SC group, LUT50 treatment increased the levels of GAP (~1.26-fold) and NADPH (~1.88-fold), while decreasing DF6P (~0.81-fold), DHAP (~0.78-fold), E4P (~0.75-fold), α-KG (~0.73-fold), fumarate (~0.65-fold), and succinate (~0.51-fold) (Figure 4A). In amino acid metabolism, serine (~1.22-fold) and glutamine (~1.13-fold) were increased, whereas glutamate (~0.82-fold), aspartate (~0.80-fold), phenylalanine (~0.79-fold), valine (~0.74-fold), and GSH (~0.63-fold) were decreased compared with the SC group (Figure 4B). Overall, LUT50 treatment was associated with distinct alterations in metabolic intermediates related to glucose, TCA-cycle, and amino acid metabolism.

To assess epigenetic changes, the expression of genes encoding histone-modifying enzymes was examined. LUT50 significantly reduced the expression of the histone methyltransferase genes *Ash1* (*p* < 0.001) and *Set1* (*p* < 0.05), whereas no significant differences were observed for the histone acetyltransferase *nej* or the deacetylases *Sir2* and *HDAC4* (Figure 4C). Despite these limited transcriptional changes, immunoblot analysis revealed significantly elevated levels of H3K36me3, H3K9ac, and H3K27ac following LUT50 treatment (*p* < 0.05; Figures 4D,E). These findings demonstrate that LUT50 treatment is associated with coordinated alterations in metabolic profiles and histone modification patterns in adult female flies.

### 3.5 Luteolin accelerates developmental progression and alters endocrine profiles during *Drosophila melanogaster* development

**Figure 5:**
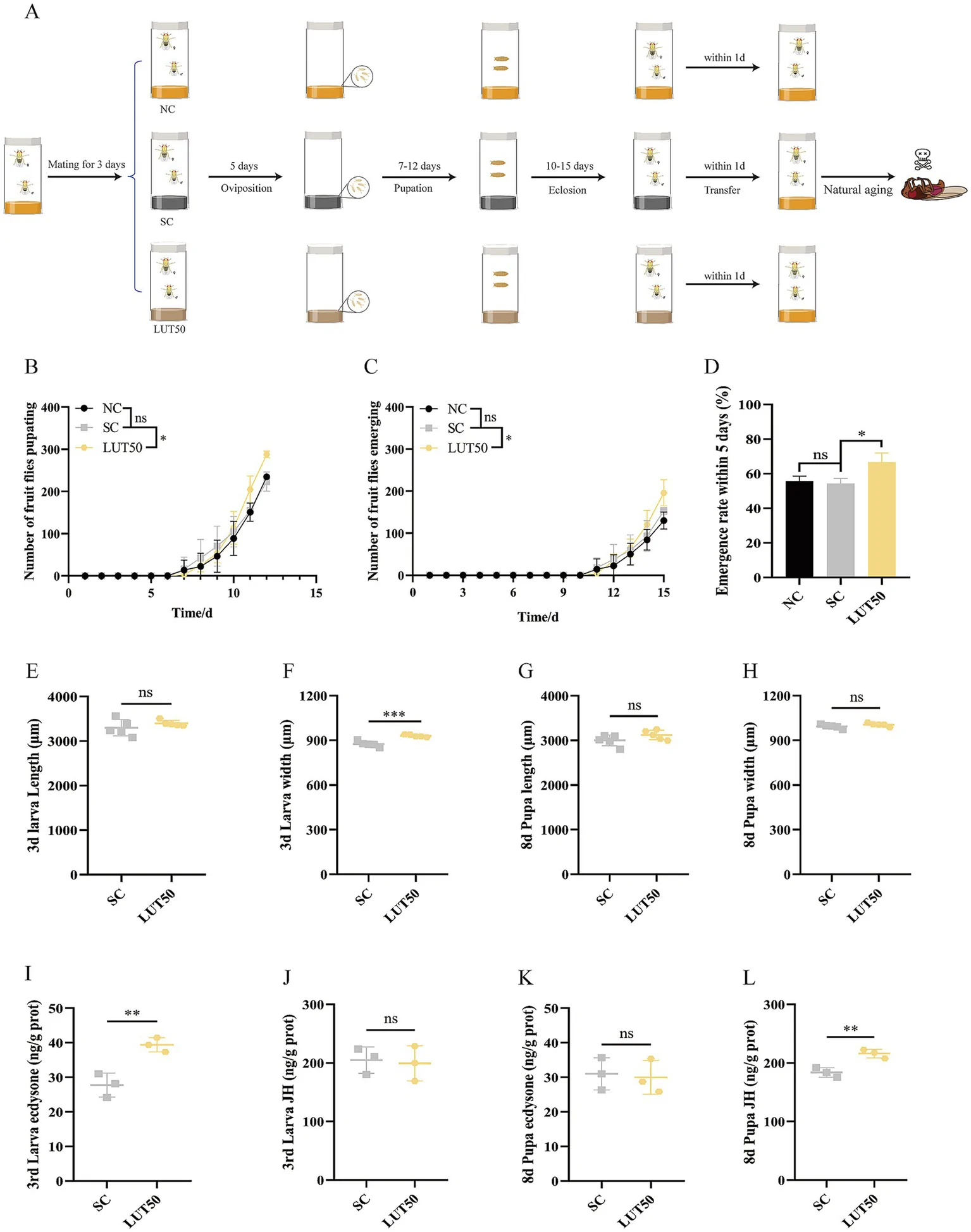
Effects of luteolin intervention during embryonic development on *D. melanogaster* development. **(A)** After 3 days of mating (3 males + 3 females), flies were transferred to specified diets for 5 days of egg laying. Eggs were monitored daily for development, and newly eclosed progeny (< 8 h) were transferred to standard diet (60 flies per group; 10 females and 10 males per vial, 3 vials). Mortality was recorded daily under natural aging. **(B)** Number of pupae formed within 12 days. **(C)** Number of adults emerged within 15 days. **(D)** Emergence rate within 5 days. **(E)** Body length of third-instar larvae. **(F)** Body width of third-instar larvae. **(G)** Body length of 8-day-old pupae. **(H)** Body width of 8-day-old pupae. **(I)** Ecdysone levels in third-instar larvae. **(J)** Juvenile hormone levels in third-instar larvae. **(K)** Ecdysone levels in 8-day-old pupae. **(L)** Juvenile hormone levels in 8-day-old pupae.

To determine whether luteolin exerts stage-dependent effects, its impact during embryonic development was investigated. As shown in Figure 5A, male and female flies were co-cultured in NC, SC, and LUT50 media for 5 days to allow egg laying. Developmental progression, morphology, and endocrine status were subsequently evaluated, and the lifespan of emerged adults was assessed under NC conditions.

Compared with the SC group, no significant differences in pupation or eclosion were observed in the NC group. In contrast, LUT50 treatment significantly increased the number of pupae within 12 days, the number of eclosed adults within 15 days, and the cumulative emergence rate during the first 5 days (Figures 5B–D). LUT50 did not affect the length of third-instar larvae but significantly increased larval width (*p* < 0.001). No significant differences were observed in pupal length or width at day 8 (Figures 5E–H). Because ecdysone and juvenile hormone are key regulators of *Drosophila* development, their levels were further determined. LUT50 treatment significantly increased ecdysone levels (~1.42-fold) in third-instar larvae (*p* < 0.01) without affecting juvenile hormone levels. In contrast, juvenile hormone levels were significantly elevated (~1.18-fold) in 8-day-old pupae (*p* < 0.01), whereas ecdysone levels remained unchanged (Figures 5I–L).

Together, these results indicate that embryonic luteolin exposure accelerates developmental progression and is associated with distinct hormonal changes during larval and pupal stages.

### 3.6 Embryonic luteolin exposure shortens adult lifespan and alters aging-related pathways

**Figure 6:**
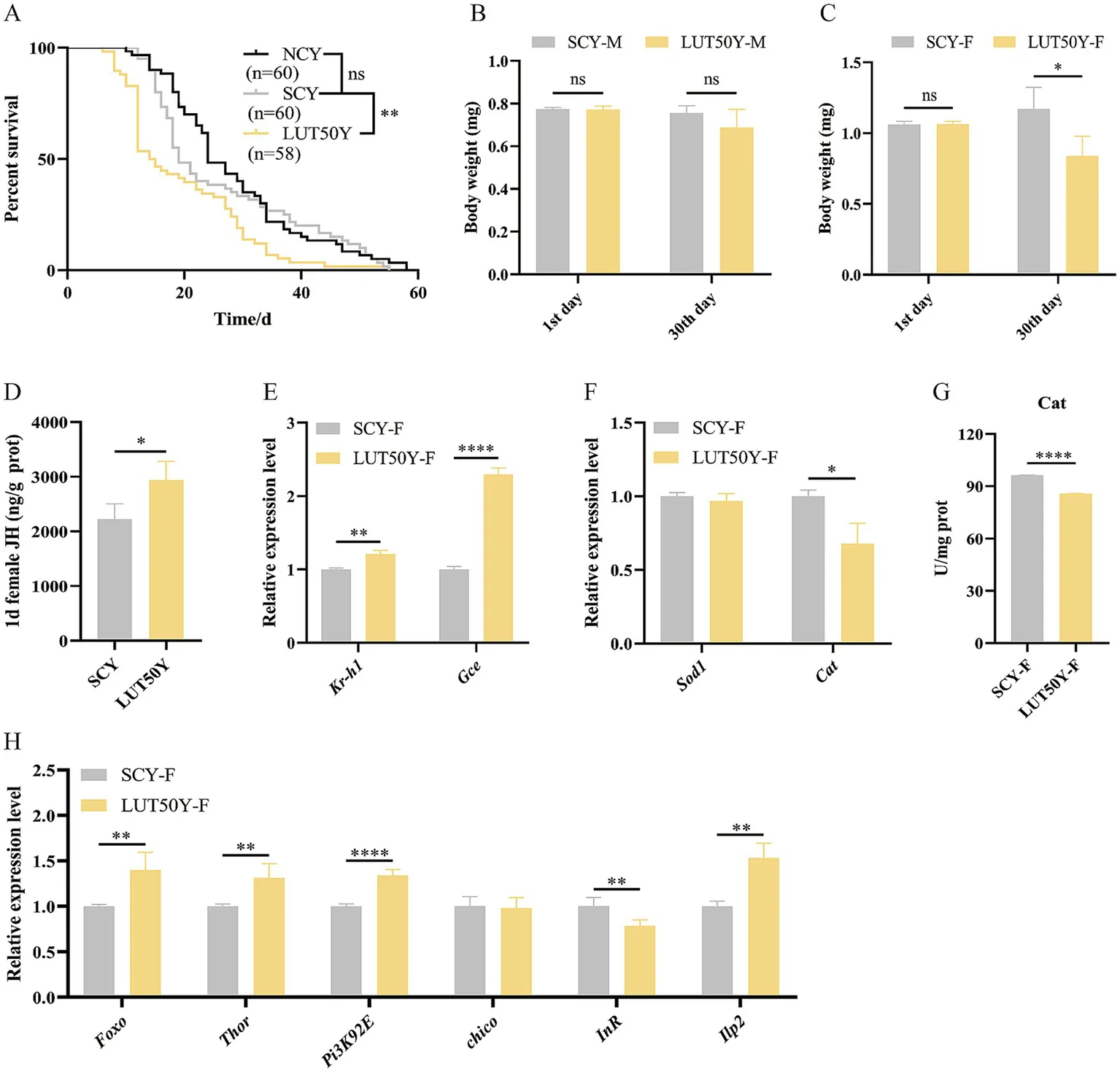
Effects of embryonic luteolin intervention on natural aging in *D. melanogaster*. **(A)** Lifespan of adult flies under natural aging after eclosion on standard medium. **(B)** Body weight of male flies at day 1 and day 30. **(C)** Body weight of female flies at day 1 and day 30. **(D–H)** In 1-day-old female adults: **(D)** Juvenile hormone (JH) levels, **(E)** expression of JH-related genes, **(F)** Expression of antioxidant enzyme genes, **(G)** CAT activities, and **(H)** Expression of insulin signaling–related genes. Kaplan–Meier survival curves were analyzed using the log-rank (Mantel–Cox) test. Statistical significance is indicated as follows: Ns, not significant; *p < 0.05; **p < 0.01; ***p < 0.001; ****p < 0.0001.

To evaluate the long-term consequences of embryonic luteolin exposure, lifespan and aging-related parameters were assessed in adult flies derived from LUT50-treated embryos. No significant differences in lifespan were observed between the NC and SC groups. In contrast, flies exposed to LUT50 during embryonic development exhibited a significantly shortened adult lifespan compared with the SC group (*p* < 0.01; Figure 6A).

Body weight analysis revealed no significant differences in males at either day 1 or day 30, and no differences in females at day 1. However, female body weight was significantly reduced at day 30 following embryonic LUT50 exposure (*p* < 0.05) (Figures 6B,C). To further investigate potential physiological alterations, hormone levels, antioxidant status, and insulin signaling were examined in 1-day-old female flies derived from LUT50-treated embryos. Compared with the SC group, juvenile hormone levels were significantly increased by 1.32-fold (*p* < 0.05) (Figure 6D), accompanied by elevated expression of the juvenile hormone-responsive genes *Kr-h1* (*p* < 0.01) and *Gce* (*p* < 0.0001) (Figure 6E). With respect to antioxidant defense, *Sod* expression remained unchanged, whereas *Cat* expression (*p* < 0.05) and catalase activity (*p* < 0.0001) were significantly reduced (Figures 6F,G). In addition, embryonic LUT50 exposure significantly increased the expression of *Foxo* (*p* < 0.01), *Thor* (*p* < 0.01), *Pi3K92E* (*p* < 0.0001), and *Ilp2* (*p* < 0.01), while reducing *InR* expression (*p* < 0.01) (Figure 6H). Together, these findings demonstrate that embryonic luteolin exposure results in persistent alterations in endocrine regulation, antioxidant defense, and insulin signaling in adult female flies.

## 4 Discussion

In this study, we investigated whether the anti-aging effects of luteolin are dependent on biological context under physiological aging conditions using *D. melanogaster* as a model organism. Specifically, we focused on two key biological variables, sex and developmental stage, to determine how they influence the longevity-promoting effects of luteolin. Through integrative analyses of lifespan, healthspan-related phenotypes, transcriptomics, metabolomics, and epigenetic regulation, we characterized the molecular responses associated with luteolin intervention. Unlike previous studies, which have primarily focused on the pharmacological or disease-protective effects of luteolin, the present study was designed to determine how biological context influences its effects on physiological aging. The findings demonstrate that the anti-aging effects of luteolin in *Drosophila* are highly dependent on biological context, with biological sex and developmental stage representing two critical determinants of its efficacy. Adult supplementation extended lifespan and improved healthspan in females but produced little benefit in males, whereas embryonic exposure resulted in shortened adult lifespan. These contrasting outcomes indicate that the biological effects of luteolin in *Drosophila* are context dependent rather than universally beneficial. To our knowledge, this is the first study to systematically compare embryonic and adult luteolin exposure within the same experimental framework while integrating multi-omics analyses to elucidate the underlying mechanisms. Collectively, these findings not only provide new evidence supporting the antiaging potential of luteolin in *Drosophila* but also highlight the importance of considering biological context, particularly sex and developmental stage, when evaluating dietary flavonoids as nutritional interventions for healthy aging.

*Drosophila melanogaster* is a powerful model for aging research, as its short lifespan and conserved molecular pathways enable the rapid identification of mechanisms relevant to higher organisms. Despite differences from humans in physiology and organ complexity, fruit flies share remarkable conservation in key signaling pathways, including the insulin/IGF, TOR, sirtuin, and stress response pathways. Importantly, males and females exhibit marked differences in morphology, physiology, behavior, and aging processes. Such sexual dimorphism is not only a fundamental biological characteristic but also a critical factor that must be considered in studies of aging and lifespan regulation (22). Among natural compounds with potential antiaging effects, luteolin, a flavonoid widely found in fruits, vegetables, and medicinal plants, has attracted attention due to its antioxidant, anti-inflammatory, and neuroprotective properties, all of which are highly relevant to aging-related decline (23). In our study, luteolin was administered at 50 μg/mL (~175 μM) in the culture medium (24). The selection of this concentration was based on both published literature and our previous experimental experience. Previous *Drosophila* studies have generally employed luteolin concentrations ranging from 5 to 100 μM, while dietary supplementation studies in rodents have commonly used doses of 50–300 mg/kg (25, 26), indicating that the concentration used here falls within the range of pharmacologically active doses reported in experimental models. In our previous study (27), we systematically evaluated three concentrations of resveratrol (10, 50, and 100 μg/mL) and found that 50 μg/mL consistently produced the greatest improvements in lifespan and healthspan without detectable toxicity. Based on this optimization experience, we selected 50 μg/mL as a representative biologically active concentration for evaluating the anti-aging effects of luteolin in the present study. Nevertheless, we acknowledge that the use of a single concentration represents a limitation of this work. A systematic dose–response analysis will be included in future studies to determine the optimal therapeutic window and further strengthen the translational relevance of our findings. Reported human oral pharmacokinetic studies typically show sub-micromolar plasma exposures (peak plasma concentration of luteolin is on the order of ~26.25–248.79 ng/mL), largely as glucuronide/sulfate conjugates (28). Thus, our working concentration is ~ 200–1900-fold above typical human systemic peak plasma concentration. Note that dietary concentration does not equal hemolymph/plasma concentration in flies; conversely, transient intestinal concentrations in humans can exceed plasma levels, and conjugates may deconjugate locally. Therefore, our dose should be interpreted as a pharmacological upper-bound test rather than a direct simulation of human exposure. This distinction should be considered when interpreting the translational significance of our findings. These pharmacokinetic characteristics may partially explain why the concentrations required to elicit robust biological effects in experimental models are substantially higher than the plasma concentrations typically achieved following dietary intake in humans. Extensive first-pass metabolism and rapid glucuronidation/sulfation markedly reduce the circulating levels of free luteolin, thereby limiting its bioavailability at target tissues despite its potent biological activities observed *in vitro* and in animal models (29). Consequently, improving the pharmacokinetic profile of luteolin is considered a prerequisite for its successful clinical translation as a antiaging agent. Recent advances in nanoformulations, liposomal delivery systems, phospholipid complexes, and polymeric nanoparticles have shown considerable promise in enhancing the solubility, metabolic stability, and systemic bioavailability of luteolin, thereby improving its pharmacological efficacy *in vivo* (30, 31). Therefore, although the present study demonstrates the anti-aging potential of luteolin in *Drosophila*, future studies should combine optimized delivery strategies with validation in mammalian models to better evaluate its translational potential.

The findings reveal that luteolin treatment in adult female flies not only extended median and maximum lifespan but also promoted health span, as evidenced by improved locomotor performance and enhanced resistance to oxidative stress, while body weight remained largely unchanged during adulthood. Previous reports have shown that reduced body weight from early to mid-adulthood was associated with slower biological aging, while improvements in locomotor and stress resistance capacities contribute to the maintenance of health span (32–34). Phenotypic changes in offspring can sometimes be influenced by maternal environmental factors, known as maternal effects (35). However, we did not observe significant changes in the lifespan of progeny derived from luteolin-exposed females, suggesting that the anti-aging benefits of luteolin act directly on exposed females and are not transmitted to the next generation. In contrast to females, male flies showed no lifespan extension under the same intervention conditions. This observation corroborates the findings of Lashmanova et al. (36) and strengthens the evidence that the anti-aging effects of luteolin are distinctly sex-dependent. A possible explanation lies in reproductive–metabolic trade-offs: in females, energy allocation during lifespan trajectories is constrained by oocyte formation, making them more responsive to luteolin that extend longevity. Males, by contrast, have lower reproductive costs and reduced metabolic plasticity, which may limit their capacity to translate luteolin exposure into lifespan gains (37). Interestingly, this gender-selective effect aligns with previous findings on rapamycin, an mTOR kinase inhibitor, which also exhibited gender-dependent effects on lifespan (38). Moreover, luteolin produced robust benefits after a 10-day intervention, without requiring long-term accumulation. Similar observations have been reported in cross-species studies, where short-term rapamycin administration during early adulthood yielded lifespan benefitted comparable to lifelong treatment in both flies and mice (39). The shared features of luteolin and rapamycin, gender specificity and durable effects following short-term intervention, suggest that their mechanisms of action may partially overlap.

Mechanistic analyses revealed that luteolin exerts its anti-aging effects in adult female flies through a multi-target regulatory network. In the antioxidant system, luteolin significantly upregulated *CncC* and *Cat* expression and enhanced Cat enzymatic activity. Unlike conventional antioxidant strategies that rely on exogenous free radical scavenging, activation of the CncC/Nrf2 homolog strengthens endogenous antioxidant defenses, thereby delaying functional decline and maintaining homeostasis (40). Cat decomposes hydrogen peroxide, helping to preserve mitochondrial redox balance and protect cells from oxidative damage (41). Moreover, luteolin inhibited the IIS/PI3K–mTOR pathway, while simultaneously activating AMPK- and MAPK-related pathway genes. Concomitant inhibition of IIS/PI3K–mTOR and activation of AMPK/MAPK pathways facilitates energy sensing, autophagy, and stress adaptation—key molecular hubs underlying longevity regulation (42, 43). Notably, the inhibitory effect of luteolin on mTOR mirrors the mechanism of rapamycin, further underscoring their mechanistic convergence. Luteolin also upregulated autophagy-related genes (*Atg5* and *Atg8a*) and immune regulators (*Dif* and *Relish*), indicating enhanced autophagy and immune homeostasis. Autophagy, which clears damaged organelles and protein aggregates, has been validated as a key determinant of longevity, while immune balance is equally indispensable for delaying aging (44–46). Together, these findings suggest that luteolin promoted anti-aging effects through coordinated regulation of multiple homeostatic mechanisms. Metabolic stress responses, autophagy, and immune regulation are fundamental processes that maintain cellular homeostasis and organismal health during aging (47, 48). Moreover, restriction of nutrient-sensing signaling and improvement of metabolic quality are widely recognized mechanisms of lifespan extension. Increasing evidence indicates that metabolic intermediates are not merely substrates for energy production but can also function as signaling molecules that influence chromatin states and gene expression through epigenetic modifications, thereby reinforcing pro-longevity transcriptional programs (49). Therefore, the coordinated changes observed in nutrient-sensing pathways, autophagy, immunity, metabolism, and epigenetic regulation may collectively contribute to the lifespan-promoting effects observed in *Drosophila* following luteolin treatment.

Metabolic profiling provided further evidence: LUT50 treatment enhanced the pentose phosphate pathway, redirecting glucose flux toward NADPH production, thereby increasing cellular reductive capacity and reducing mitochondrial oxidative load (50). Concurrently, glutamine and serine levels were elevated, while TCA intermediates decreased. Previous studies have shown that supplementation with glutamine or serine can extend lifespan, while reduced TCA intermediates help limit mitochondrial stress and ROS generation (51, 52). Thus, the metabolic changes induced by luteolin in *Drosophila* are consistent with a “longevity-associated” metabolic state characterized by reduced mitochondrial burden and enhanced antioxidant capacity. At the epigenetic level, luteolin reduced transcription of histone methyltransferases Ash1 and Set1, with no significant changes in histone acetylation regulator gene (*nej*, *Sir2*, *HDAC4*). Nevertheless, immunoblotting revealed elevated levels of H3K36me3, H3K9ac, and H3K27ac. H3K36me3 has been implicated in promoting transcriptional fidelity and lifespan extension (53); H3K9ac, while increasing with age, enhances the expression of antioxidant and mitochondrial genes when elevated at younger stages (54); and H3K27ac, as an enhancer activation mark, is closely linked to immune regulation (55). These results suggest that luteolin may promote a transcriptionally permissive epigenetic state in Drosophila, possibly through altered enzyme activity, recruitment, or metabolite availability, thereby facilitating the expression of genes involved in metabolism, stress responses, and immunity. Taken together, these findings support a model in which nutrient-sensing regulation, metabolic remodeling, and epigenetic reprogramming may act in concert to contribute to the lifespan-promoting effects of luteolin in *Drosophila*.

Unlike the lifespan-promoting effects observed following adult supplementation, embryonic exposure to luteolin shortened adult lifespan in *Drosophila*. LUT50 exposure significantly accelerated key developmental processes, including pupation and eclosion, and markedly shortened the natural lifespan of adult flies. These developmental and lifespan alterations may be mechanistically linked, potentially mediated by hormonal pathways such as ecdysone and juvenile hormone signaling, which play central roles in regulating both metamorphosis and aging (56, 57). LUT50 intervention alters endocrine homeostasis across developmental stages, as evidenced by elevated ecdysone in larvae and increased JH levels in pupae and 1-day-old female adult. In addition, JH-regulated genes (*Kr-h1*, *Gce*) were upregulated, antioxidant gene *Cat* and Cat activity were reduced in 1-day-old female adult. JH is a critical endocrine regulator of metamorphosis, reproduction, and aging in insects, known to promote reproduction at the cost of lifespan (58). These findings suggest that embryonic luteolin exposure may disrupt endocrine and antioxidant homeostasis in adult female *Drosophila*, thereby contributing to the shortened lifespan observed in this study. Moreover, aberrant regulation of IIS pathway genes was observed: *Foxo*, *Thor*, *Pi3K92E*, and *Ilp2* were upregulated, while *InR* was downregulated. The IIS pathway coordinates metabolism, growth, stress responses, and lifespan (59). Upregulation of *Ilp2* and *Pi3K92E* suggests hyperactivation of IIS signaling, whereas downregulation of *InR* and upregulation of *Foxo* and *Thor* may reflect compensatory transcriptional feedback. Nevertheless, these adjustments appeared insufficient to functionally counteract IIS overactivation, culminating in reduced lifespan. These observations are consistent with the Developmental Origins of Health and Disease (DOHaD) hypothesis, which proposes that early-life nutritional or environmental exposures can reprogram metabolism and signaling pathways, thereby influencing adult health outcomes (60). Collectively, these findings suggest that the biological effects of luteolin are highly dependent on developmental stage in *Drosophila*. Adult supplementation promoted lifespan, whereas embryonic exposure shortened adult lifespan, indicating that the timing of intervention is a critical determinant of its biological effects. Although further validation in mammalian systems is required, our findings highlight the importance of considering developmental stage when designing and interpreting studies of dietary flavonoids in aging research.

Although the anti-aging and metabolic regulatory effects of luteolin have been reported in several rodent studies, these investigations have largely focused on disease models or adult interventions (61, 62). In contrast, the present study suggests that the biological effects of luteolin on aging are highly dependent on both developmental stage and biological sex under physiological aging conditions in *D. melanogaster*. By systematically comparing embryonic and adult exposure within the same experimental framework and integrating lifespan phenotyping with transcriptomic, metabolomic, and epigenetic analyses, our study extends current knowledge beyond simply demonstrating the lifespan-promoting effects of luteolin in this model. Instead, it identifies the biological contexts in which luteolin is more likely to exert beneficial effects and provides mechanistic insights that may guide future validation in mammalian models and facilitate the rational design of stage-specific and sex-informed nutritional strategies for healthy aging.

Nonetheless, several limitations should be acknowledged. First, only a single dose of luteolin was tested, and its dose-dependency remains to be systematically evaluated. Second, the mechanistic insights presented here are primarily correlative. Future studies employing gene editing and enzymatic activity assays are required to establish causal relationships. Third, mechanistic analyses were performed only in female flies because significant lifespan extension and healthspan improvement were observed exclusively in females. Future studies should investigate whether the molecular responses to luteolin differ between males and females to better elucidate the mechanisms underlying the observed sexual dimorphism. Finally, although *Drosophila* shares highly conserved nutrient-sensing and longevity pathways with mammals, further validation in mammalian models will be essential to determine whether the stage-dependent and sex-specific mechanisms identified in this study are conserved across species and can ultimately be translated into personalized nutritional interventions for healthy aging.

In conclusion, this study provides the first systematic evidence of the anti-aging effects of luteolin in *Drosophila* and elucidates its underlying molecular basis, highlighting its gender-specific and stage-dependent actions. During adulthood, luteolin intervention in females extended lifespan and improved health status through enhanced antioxidant defense, activation of autophagy, metabolic reprogramming, and epigenetic regulation. In contrast, premature exposure during embryogenesis accelerated aging, likely *via* hormonal imbalance and remodeling of insulin signaling. Collectively, our findings demonstrate that the biological effects of luteolin in *Drosophila* are highly dependent on biological context, particularly sex and developmental stage, highlighting the importance of considering these factors when developing nutritional strategies for healthy aging.

## Data Availability

The raw data supporting the conclusions of this article will be made available by the corresponding author upon request.
